# Radiotherapy Technique Determines the Magnitude and Persistence of T-Lymphocyte DNA Damage

**DOI:** 10.3390/cancers18162536

**Published:** 2026-08-07

**Authors:** Zsuzsa S. Kocsis, Péter Ágoston, Gyöngyi Farkas, Gábor Székely, Gyöngyvér Orsolya Sándor, Kliton Jorgo, László Gesztesi, Tibor Major, Csilla Pesznyák, András Herein, Gábor Stelczer, Dalma Mihály, Georgina Fröhlich, Zoltán Takácsi-Nagy, Csaba Polgár, Zsolt Jurányi

**Affiliations:** 1 Department of Clinical Radiobiology and Onco-Cytogenetics and The National Tumor Biology Laboratory, Centre of Radiotherapy, National Institute of Oncology, 1122 Budapest, Hungary; kocsis.zsuzsa@oncol.hu; 2 Department of Radiotherapy, Semmelweis University, 1085 Budapest, Hungary; 3 Centre of Radiotherapy and the National Tumor Biology Laboratory, National Institute of Oncology, 1122 Budapest, Hungary; 4 Department of Clinical Radiobiology and Onco-Cytogenetics, Centre of Radiotherapy, National Institute of Oncology, 1122 Budapest, Hungary; 5 Department of Clinical Oncology, Semmelweis University, 1085 Budapest, Hungary; 6 Centre of Radiotherapy, National Institute of Oncology, 1122 Budapest, Hungary

**Keywords:** T-lymphocyte damage, DNA damage, chromosome aberrations, immune cell dose, brachytherapy, CyberKnife

## Abstract

Background: There are limited data comparing the effect of different radiotherapy techniques on healthy cells in the same patient group. Furthermore, assessing radiotherapy-induced T-lymphocyte damage may be important given the increasing use of immunotherapy. We aimed to measure and compare DNA damage in T-lymphocytes after four types of radiotherapy for low- and intermediate-risk prostate cancer and monitor their persistence for five years. Methods: A prospective comparison of patients receiving conventional LINAC (linear accelerator) (70–78 Gy), CyberKnife teletherapy (37.5–40 Gy), low-dose-rate brachytherapy (LDR; 145 Gy) and high-dose-rate brachytherapy (HDR; 19–21 Gy was performed using the chromosome aberration technique (at 3, 6, 9, 12, 24, 36, 48, and 60 months, 192 patients). Multivariate regression analyses were conducted to assess the predictive potential of chromosome aberrations for toxicities. Results: We found that teletherapy techniques (conventional LINAC and CyberKnife therapy) caused 1.6–3.6-fold more chromosomal aberrations than brachytherapy. At three months, 4.4–13.1% of T-lymphocytes were damaged. Five years after treatment, the total aberration values of conventional LINAC and LDR brachytherapy patients were still significantly higher than those before treatment (*p* < 0.001 for LINAC and *p* = 0.011 for LDR). Significant regression models suggested that total aberrations or aberrant cell frequency might predict toxicities in addition to the biologically effective dose (BED) and the irradiated volume (V_100%_) (*p *= 0.020 for the model including total aberrations, *p* = 0.010 for the model including aberrant cell frequency). Conclusions: We observed a lower chromosome aberration frequency and fewer toxicities in brachytherapy patients. We also demonstrated that long-term T-lymphocyte damage depends on the type of radiotherapy.

## Simple Summary

We compared DNA damage in immune cells after four different types of prostate radiotherapy and found significant differences between radioactive isotope-based and machine- based radiotherapy. The proportion of damaged immune cells called T-lymphocytes was 4.4–13.1% at three months. There is a possibility that immunotherapy after radiotherapy will not be as effective. However, choosing radioactive isotope-based radiotherapy instead of machine-based help preserve immune cell function. In some treatments, we found that immune cell damage was still present five years after treatment. We described how patient characteristics and radiotherapy-related factors affect this immune cell damage. Our findings also suggest that, these level of immune cell damage may predict the risk of side effects.

## 1. Introduction

Approximately half of all patients with tumors are treated via different radiotherapy techniques [1]. Treatment modalities and fractionation schedules are commonly evaluated using the linear–quadratic model to describe dose–response relationships and via biologically effective dose (BED) calculations to compare different fractionation schemes. However, the relative contribution of treatment parameters, for example, radiation energy, fractionation, overall treatment time and dose rate to the overall effect on healthy cells remains uncertain, limiting deeper mechanistic understanding [2].

Therefore, our primary aim was to measure the chromosome aberrations after different radiotherapy techniques in the same patient cohort.

As low- and intermediate-risk prostate cancer is one of the few tumor types that can be routinely treated with multiple different radiotherapy techniques, we chose this patient group for our investigations. In our institute, teletherapy can be delivered by conventional linear accelerators (LINACs) or by a CyberKnife LINAC with the precision of its robotic arm. Greater conformality allows higher doses per fraction and shorter overall treatment times. In contrast, brachytherapy employs radioactive isotopes implanted into the tumor. Tumor movement is less problematic in this setting, and smaller safety margins can be applied around the clinical target volumes (CTVs) (to account for organ motion). A high dose gradient also helps to prevent normal tissue toxicity. We compared the established biological dosimetry parameters, namely chromosome aberration frequencies, following treatment with these four radiotherapy modalities.

On the other hand, radiation-induced DNA damage in chromosome aberration method is commonly quantified using T-lymphocytes as surrogate markers of healthy tissue [3]. Therefore, it also describes the condition of immune cells. Phytohemagglutinin (PHA) induces only T-lymphocytes proliferation in peripheral blood cultures. Therefore, with the chromosome aberration method, we can measure T-lymphocyte DNA damage. These data can be important, because it was previously shown by others that DNA damage was shown to cause T-cell senescence [4,5,6], which was demonstrated to be a factor in the success rate of immunotherapy [7,8,9,10].

Our secondary goal was to compare DNA damage in T-lymphocytes measured as chromosome aberrations between modalities. Although immunotherapy is not a major treatment modality for prostate cancer, this patient group may be a suitable model as multiple techniques are used to treat this population.

Chromosome aberrations are also suggested as surrogate markers to predict radiotherapy toxicities (without being causative factors) [11,12,13] for which no universally accepted standard method exists in clinical practice. We aimed to test this hypothesis by comparing chromosome aberrations and graded genitourinal and gastrointestinal radiation toxicities.

Furthermore, there is also scarce information on how long it takes to eliminate damaged T-cells [14], and we planned to monitor the persistence of aberrations for five years.

## 2. Patients and Methods

### 2.1. Patients

Between April 2015 and June 2021, low- and intermediate-risk prostate cancer patients [15] (N = 192) were enrolled in our study (Table 1). Patients with previous tumors were excluded. Blood samples were taken before treatment and during follow-up visits, when a PSA (prostate-specific antigen) measurement was also performed. The toxicities were evaluated with the validated EORTC-RTOG grading system, a developed for this purpose by the European Organization for Research and Treatment of Cancer–Radiation Therapy Oncology Group. The toxicity endpoints were determined as maximum grades, with every patient counted once. Additionally, the International Prostate Symptom Score (IPSS) and the quality of life (QoL) questionnaires were completed by the patients. Blood samples were taken once from healthy men (N = 22) whose age matched to the patient group.

### 2.2. Radiotherapy

At our institute, four types of radiotherapy are available for the treatment of non-metastatic prostate cancer.

Brachytherapy techniques are recommended for patients whose prostate measures less than 60 cm^3^, whose rectum–prostate distance is greater than 5 mm, and with no pubic arch interference. Patients with an initial IPSS greater than 15 were advised against brachytherapy because that time one-fraction high-dose-rate (HDR monotherapy and low-dose-rate (LDR) brachytherapy were administered in a randomized clinical study (NCT02258087) in our center. For patients without these contraindications, patient preference for teletherapy or brachytherapy was considered. The dose-volume constraints are listed in Supplementary Table S1. The CyberKnife system was installed at our institute in 2018. Before that, all teletherapy patients received LINAC therapy. After the installation, patients could also opt for CyberKnife therapy.

LINAC therapy: For intermediate-risk patients, the prostate CTV was expanded by a 0.5 cm margin (except in the posterior direction, where it was not extended), and a 1.0 cm margin was added cranially for seminal vesicle irradiation. Low-risk patients received 70–78 Gy to the prostate, and intermediate-risk patients received 57.4–60 Gy to the seminal vesicles. Both simultaneous integrated boost (SIB: 2.5 Gy/fraction on the prostate, 2.05 Gy/fraction on the seminal vesicles) and classical (2 Gy/fraction) fractionation were applied [16]. Treatment planning was performed using Eclipse v13.7 (Varian, Palo Alto, CA, USA) or Pinnacle v9.8 (Philips, Eindhoven, The Netherlands) systems. The dose-volume constraints are shown in Supplementary Table S1.

Cyberknife therapy: For a precise setup, four gold markers were inserted into the prostate two weeks prior to the planning CT. 37.5–40 Gy dose was prescribed to the prostate, and for intermediate-risk patients, 30–32.5 Gy was also given to the seminal vesicles CTV. Radiotherapy was delivered using a CyberKnife system (Accuray, Madison, WI, USA) in five fractions, which were administered every second working day [17].

LDR brachytherapy (seed BT): Low-dose-rate brachytherapy was performed with stranded ^125^I seeds (BEBIG, Berlin, Germany). After anesthesia, catheters were inserted into the prostate with rectal ultrasound guidance in the lithotomy position, and the seeds were implanted (SPOT PRO 3.1 and Oncentra Prostate 3.2.2). A total of 145 Gy was delivered on the surface of the prostate [18,19].

HDR brachytherapy: After Foley catheter insertion, transrectal ultrasound-guided needle insertion was performed, and an intraoperative plan was made (Oncentra Prostate 3.2.2). The dose delivery was executed with an afterloading machine (Elekta Brachytherapy, Veenendaal, The Netherlands), and the prescribed dose was 19 or 21 Gy (^192^Ir) [20].

### 2.3. Baseline Characteristics

#### T-Lymphocyte Sample Preparation and Chromosome Analysis

Blood samples were collected by venipuncture into Li-heparinized tubes. T-lymphocytes were induced to proliferate with phytohemagglutinin M (2% *v*/*v*, Gibco, Grand Island, NY, USA, cat: 10576015) in supplemented (15% fetal bovine serum, 100 U/mL penicillin, 100 mg/mL streptomycin) RPMI 1640 (Roswell Park Memorial Institute 1640) media. Only T-lymphocytes divide when induced by PHA [21,22,23,24]. After 48 h of culture at 37 °C, colcemid (0.1 µg/mL, Gibco, cat: 15212012) was added, which inhibits the mitotic spindle and stops division in metaphase. Two hours later, the culture was centrifuged (400× *g*, 8 min, RT) and the supernatant was discarded. The pellet was treated with hypotonic KCl solution (0.075 M, 37 °C, 15 min) for T-lymphocyte swelling and red blood cell lysis, followed by centrifugation (450× *g*, 8 min, RT). The supernatant was discarded and chromosomes were fixed with 10 mL methanol: acetic acid (3:1, *v*/*v*) (repeated five times). The cell suspension was dropped onto glass slides, followed by subsequent Giemsa staining (3% Giemsa in phosphate buffer pH = 7.2). One hundred metaphases on four coded slides were analyzed at 1000× magnification.

Chromosome aberrations were analyzed in metaphases with 44–47 chromosomes, according to international standards [25]. Chromatid breaks, chromosome fragments, exchanges, translocations, dicentrics and rings were evaluated. Their sum is the total aberration value. Based on decades of practice, 5 aberrations/100 cells are considered the normal range of total aberrations in our laboratory [26]. The aberrant cell number is the number of cells with any aberration per 100 cells, which indicates the frequency of damaged cells. The samples were coded; the scoring criteria were harmonized between the scorers and consistency in equipment usage was ensured.

Chromosome aberrations were measured before treatment, immediately after treatment, and 3, 6, 9, 12, 24, 36, 48, and 60 months after radiotherapy.

### 2.4. Statistical Analysis

The distribution of Gleason score, risk, and androgen deprivation therapy use varied significantly between the groups, but no differences were observed in the T status (size and direction of tumor spread in the TNM score system representing tumor, node, and metastasis status, respectively), age, or iPSA (initial PSA before treatment) level (Table 1).

Therefore, we applied stabilized propensity score weighting in order to balance the initially imbalanced patient groups [27,28,29]. The propensity score was estimated as the probability of assignment to the treatment group received, conditional on baseline characteristics (T status, Gleason score, iPSA, risk, age, and ADT use). Based on this propensity score and the treatment group/study population ratio, every patient received a weight value (stabilized inverse probability weight) [30], which was used throughout the analysis. The initial and pseudo-population characteristics are shown in Table 1 and Table 2.

ANOVA tests were performed (Kruskal–Wallis for continuous variables) to compare the baseline characteristics of patients in different treatment arms.

Due to zero-inflation, right-skewed non-normality, and high overdispersion ratios in the raw dependent variable, traditional two-way analysis of variance (ANOVA) was statistically invalid for analysis of time and treatment dependence of chromosome frequencies, questionnaire results and PSA values. Furthermore, the experimental design featured highly unbalanced group sizes. To address these structural issues simultaneously, a two-part hurdle framework was implemented using Statsoft Statistica (v12.5). Part 1 modeled the probability of clearing the “zero hurdle” using a binary logistic regression. The dependent variable was mathematically dichotomized into zeroes and positive values. Part 2 modeled the magnitude of the positive continuous decimal outcomes by applying a General Linearized Model (GLZ) strictly to the subset of cases that cleared the zero hurdle. GLZ model was performed with gamma distribution and log-link function. The GLZ model utilized an overparameterized Type III Sum of Squares estimation to prevent unequal sample sizes from biasing the hypothesis testing. For both parts, the main effects of time, treatment type, and their two-way interaction term (time X treatment type) were evaluated. Post hoc pairwise comparisons for significant interactions were adjusted using Benjamini–Hochberg correction to protect against Type I error inflation (Supplementary Table S2).

**Figure 1:**
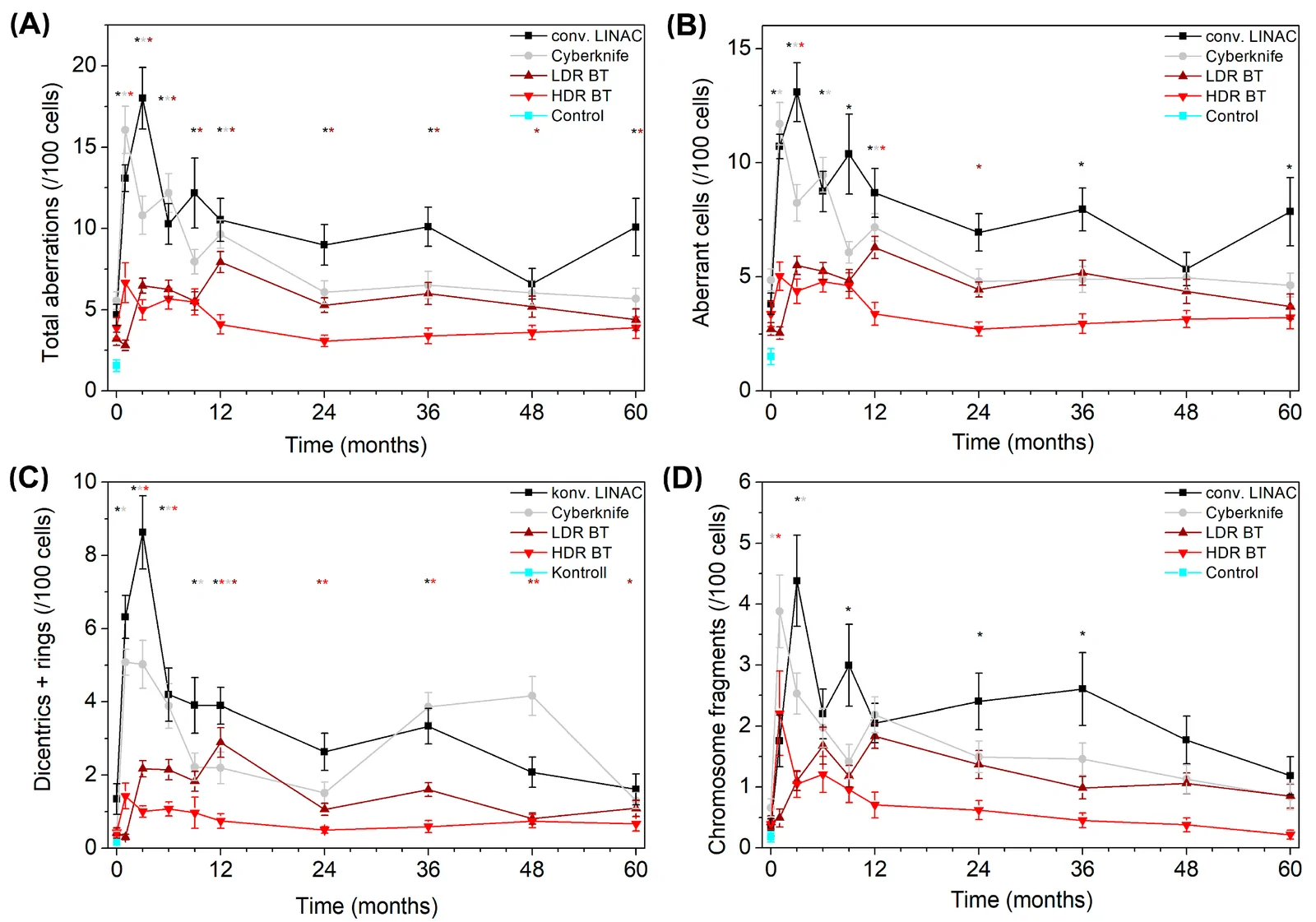
The time dependence of chromosome aberrations after conventional LINAC (linear accelerator, black) and CyberKnife (gray) therapy, LDR BT (low-dose-rate brachytherapy, burgundy), HDR BT (high-dose-rate brachytherapy, red), and age-matched controls (cyan). The significant differences compared with values before treatment of each modality are with post hoc tests and increases were indicated with asterisks * in the appropriate color. The error bars represent the SEM. (**A**) Total aberrations (**B**) Aberrant cells (**C**) Dicentrics + rings (**D**) Chromosome fragments are shown before and after the four treatment types. The value of age-matched controls (measured once) is indicated in blue.

**Figure 2:**
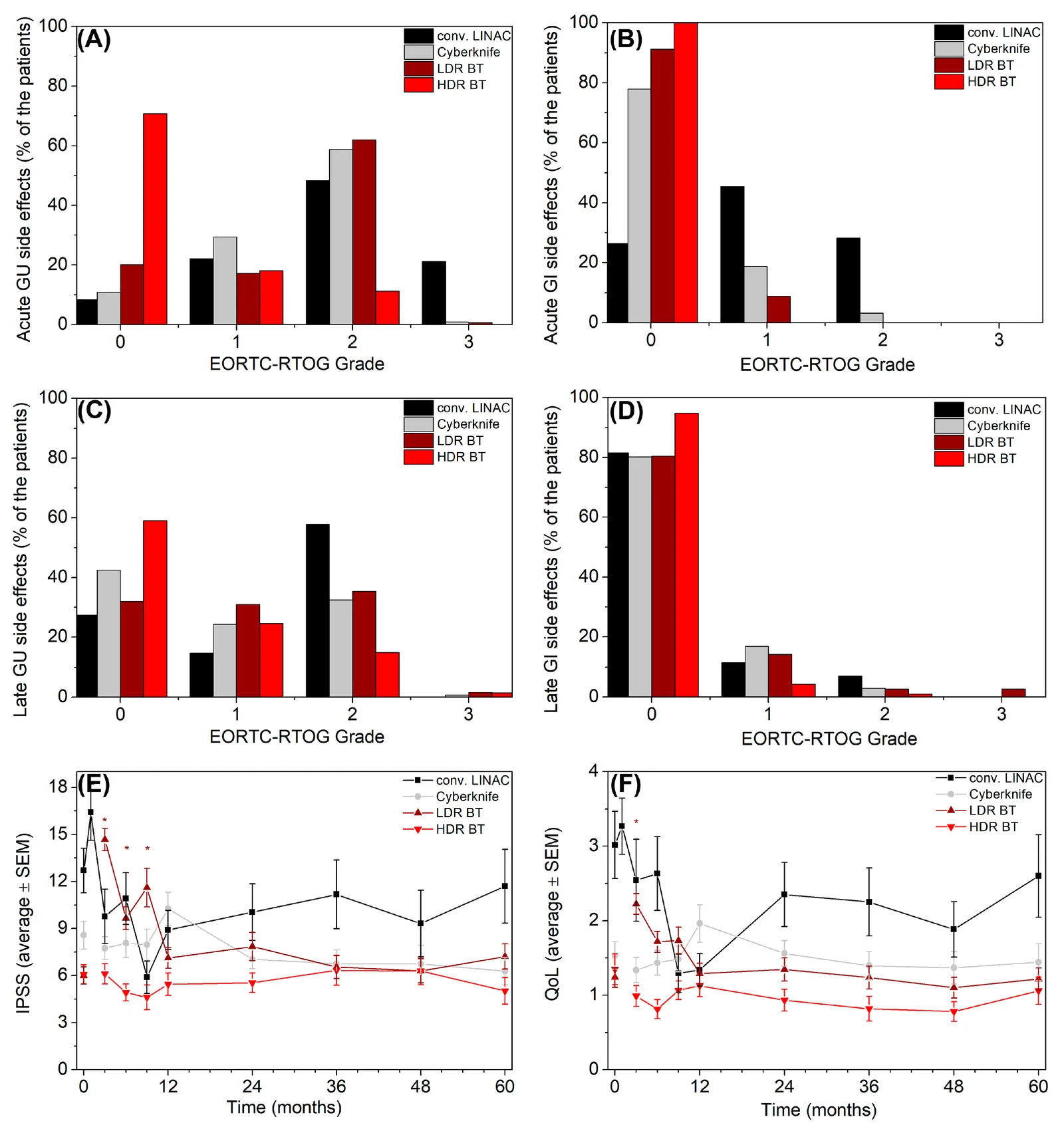
Toxicities of conventional LINAC (black), CyberKnife (gray) therapy, LDR BT (burgundy) and HDR BT (red). (**A**) Acute genitourinary toxicities (GU) (**B**) Acute gastrointestinal toxicities (GI) (**C**) Late genitourinary toxicities (GU) (**D**) Late gastrointestinal toxicities (GI) (**E**) International Prostate Symptom Score (IPSS) (**F**) Quality of Life questionnaire scores (QoL) are shown before and after the four treatment types. Significant differences compared to each modality’s pre-treatment values are indicated by asterisks * in the appropriate color. The error bars represent the SEM.

This two-part hurdle framework served a dual purpose. By evaluating the categorical factors of time and treatment, it functioned as an ANOVA-like group comparison for chromosome aberration, questionnaire results, and PSA values (Figure 1 and Figure 2, Supplementary Figure S1). On the other hand, performing the analysis per treatment type and incorporating continuous variables, it allowed for multiple regression-like predictor testing in similar models for analyzing baseline and treatment characteristics as predictors of chromosome aberrations (Supplementary Tables S3 and S4). The significant predictors in the binomial model interpreted as predictors of the presence (gatekeeper), the significant predictors of gamma model interpreted as effector of the frequency of aberrations (intensity driver).

One-sample Wilcoxon test (two-tailed with Bonferroni correction) was used to test if there was a difference between the total aberration values and the normal range of our laboratory. Multivariate linear regression analysis was applied to determine whether different chromosome aberrations immediately after radiotherapy are independent predictors of different toxicities in addition to V_100%_ (the volume irradiated with 100% of the prescribed dose) and BED (biologically effective dose) [31] (Table 2).

All tests and models were considered significant at *p* < 0.05. For data representation and analysis, StatSoft STATISTICA 12, and Origin Pro 8.5 were used.

## 3. Results

We followed 24 patients in the conventional LINAC treatment group (LINAC), 50 patients in the CyberKnife group, 66 patients in the low-dose-rate (LDR) brachytherapy group, and 52 patients in the high-dose-rate brachytherapy treatment (HDR) group. The median follow-up was 60 months in each group. After the stabilized propensity score weighting there were no differences in age, iPSA, T status, Gleason score, risk, and ADT use between the treatment groups (Table 2).

### 3.1. Changes in PSA Values After Radiotherapy

In each treatment group, the average PSA concentration in patients’ blood fell below the upper limit of the reference range of our institute (3 ng/mL). However, for HDR-treated patients, the average value was significantly higher than the values after conventional LINAC therapy at all time points for five years (Supplementary Figure S1A). Although our sample size was not sufficient to draw conclusions regarding survival, we observed lower biochemical relapse-free survival in this group (*p* = 0.003, Supplementary Figure S1B).

### 3.2. Time-Dependent Chromosome Aberrations

An increase in chromosome aberrations after irradiation was observed in all groups, but we detected 1.6–3.6 times more total aberrations in the groups receiving teletherapy (18.0 ± 1.9/100 cells for LINAC, 10.8 ± 1.2/100 cells for Cyberknife at three months after treatment) compared to the brachytherapy groups (6.5 ± 0.5/100 cells for LDR, 5.0 ± 0.6/100 cells for HDR brachytherapy) (Figure 1A). HDR therapy caused the fewest aberrations throughout our follow-up. After an initial spike, aberration frequencies began to decline, but 60 months after LDR brachytherapy and conventional LINAC treatment, the total number of aberrations still showed significant difference from pretreatment values (*p* < 0.001 for LDR brachytherapy; *p* = 0.011 for LINAC). In our laboratory, the upper limit of a negative chromosomal aberration test when screening staff at risk of radiation exposure is 5 total aberrations per 100 cells [26]. All time points after LINAC therapy (except 48 months), total aberrations were above this limit (one-sample Wilcoxon test). The mean total number of chromosome aberrations were not significantly different from this value from 24 months after CyberKnife therapy. Three, six and twelve months after LDR brachytherapy, the total number of aberrations was significantly greater than 5/100 cells. The median total number of aberrations after HDR never exceeded this threshold, not even immediately after implantation. However, the average total aberration values before HDR therapy and 24, 36, 48, and 60 months after treatment were significantly below this 5 aberration/100 cells threshold. We also plotted the average total aberrations of our twenty-two age-matched control subjects on the graphs (measured once). The total aberration value of Cyberknife therapy, LDR and HDR brachytherapy group before treatment differed significantly from the value of our healthy controls. Furthermore 60 months after HDR brachytherapy and conventional LINAC therapy the total aberration frequency was still significantly different from the value of the healthy controls.

The time dependence of the aberrant cell frequencies was similar to that of the total aberration values (Figure 1B). Three months after LINAC and CyberKnife therapy, approximately 13.1 ± 1.3% and 8.2 ± 0.8% of the T-lymphocytes had chromosome damage, respectively. Brachytherapy techniques affected fewer T-lymphocytes (5.5 ± 0.4% for LDR and 4.4 ± 0.5% for HDR therapy at three months).

Dicentric and ring aberrations (dicentrics + rings) are radiation-specific chromosome aberrations, which do not typically arise due to environmental conditions [32]. These values differed significantly from the values before treatment at every time point for twelve months after CyberKnife therapy, 36 months after conventional LINAC therapy, from twelve months to five years after LDR brachytherapy, and at 48 months after HDR brachytherapy. The dicentrics + rings values were 2.3–8.6-fold greater three months after teletherapies (8.6 ± 1.0 for conventional LINAC, 5.0 ± 0.7 for CyberKnife) than in the brachytherapy arms (2.2 ± 0.2 for LDR brachytherapy, 1.0 ± 0.2 for HDR brachytherapy). There was significant difference between the dicentrics + rings values before treatment of Cyberknife, HDR, and LDR patients and those in healthy controls (Figure 1C).

The chromosome break values showed a similar trajectory to total aberrations. These aberration values were lower after treatment but persisted for years after conventional LINAC therapy (Figure 1D).

### 3.3. The Role of Baseline and Treatment Characteristics

We assessed the effect of baseline characteristics (age, iPSA, T status, Gleason score) on aberrations using two-phase hurdle models. The risk category was omitted, as it is collinear with T status, iPSA and Gleason score, by definition (Supplementary Table S3). The most important predictor was time, as time trajectories suggested in Figure 1. Time was an independent predictor in the gamma model (intensity driver) of dicentrics + rings after conventional LINAC therapy, Cyberknife and LDR brachytherapy, as well as in the gamma model of total aberrations after Cyberknife and HDR BT. Furthermore, it was an independent predictor in binomial model (gatekeeper) in dicentrics+ rings after Cyberknife and HDR therapy. This may be because the decrease in aberrations over time eventually reaches the limit where they became undetectable. In the case of conventional LINAC therapy significant predictors were found only in the gamma stage models. Initial PSA predicted total aberrations and dicentric + ring frequencies. T stage affected the frequencies of total aberrations in this group and after Cyberknife therapy (both total aberrations and dicentrics + rings). Dicentric + ring frequency was also affected by age. Aside from time, no other predictors were found among the baseline characteristics in the LDR brachytherapy group. In the HDR brachytherapy arm only the Gleason score affected the presence of dicentrics and rings besides time.

We subsequently performed a hurdle model framework analysis that included treatment characteristics as well (Supplementary Table S4). Gleason score and T status were no longer significant independent predictors. However, iPSA remained an intensity modulator (gamma model) of both total aberrations and dicentrics + rings after conventional LINAC therapy. The appliance of the SIB technique also affected the presence of total aberrations. Additionally, seminal vesicle dose was a significant predictor of the frequency of total aberrations (gamma model) after Cyberknife therapy. In the case of brachytherapies, ADT use emerged as an independent predictor, affecting the presence of dicentrics + rings after LDR therapy, and the frequency of both total aberrations and dicentrics + rings after HDR therapy.

### 3.4. Toxicities After Different Types of Prostate Cancer Radiotherapy

EORTC-RTOG Grade ≥2 early genitourinal toxicities occurred after LINAC, CyberKnife, LDR, and HDR therapy in 69.5%, 59.7%, 62.7%, and 11.2% of patients, respectively. Of these, 21.2%, 0.9%, and 0.7% were Grade 3 toxicities (LINAC, CyberKnife, and LDR groups, respectively) (Figure 2A). There were no Grade 3 acute gastrointestinal toxicities. Furthermore, the frequency of Grade 2 early gastrointestinal effects was 28.2% after LINAC and 3.2% after CyberKnife therapy, and there were no Grade 2 toxicities after HDR or LDR brachytherapy (Figure 2B).

With respect to late genitourinal toxicities (GU), 57.8% were Grade ≥2 after LINAC, 33.2% after CyberKnife, 37.0% after LDR, 16.3% after HDR radiotherapy. The frequencies of Grade 3 toxicities were 0.7%, 1.6%, and 1.4% after CyberKnife, LDR, and HDR irradiation, respectively (Figure 2C). There was only one (2.7%) Grade 3 late gastrointestinal toxicity among the LDR brachytherapy patients, and the percentage of Grade 2 late GI (gastrointestinal) toxicities was 7.0% after conventional LINAC, 2.9% after CyberKnife therapy, 5.4% after LDR brachytherapy, and 1.0% after HDR brachytherapy (Figure 2D).

We used patient-reported questionnaires (PROMs) to assess toxicities further. The International Prostate Symptom Score questionnaire assessing urinary toxicities (score range: 0–35, where a higher score indicates worse toxicities) was completed by patients at each follow-up visit (Figure 2E). However, it was not appropriate to administer the questionnaires directly after brachytherapy and CyberKnife treatments because the questions asked about the symptoms of the past month. We measured an increase three, six, and nine months after LDR therapy (6.0 ± 0.5 to 14.7 ± 0.8 at three months), but no significant difference was found after HDR (6.1 ± 0.6 to 6.1 ± 0.6 at three months), Cyberknife (8.6± 0.9 to 7.7 ± 0.7 at three months), and conventional LINAC therapy (12.7 ± 1.4 to 9.8 ± 1.7 directly after therapy) compared with pre-treatment values.

We assessed patients’ subjective quality of life (QoL) (score range: 0–6, where a higher score indicates worse toxicities), and the scores rose three months after seed therapy (1.2 ± 0.1 to 2.2 ± 0.1), similar to the IPSS results (Figure 2F). There was no significant increase in HDR, conventional LINAC, or CyberKnife scores.

### 3.5. Connections Between Chromosome Aberrations and Toxicities

We performed a multivariate linear regression analysis to investigate the predictive power of chromosome aberrations on toxicities. We included biologically effective dose and V_100%_ (the volume that received 100% of the prescribed dose) in the models in addition to chromosome aberrations. We chose these variables to balance physical differences in treatment and test whether chromosome aberrations can represent individual radiosensitivity. Significant models were identified (Table 3): Total aberrations (*p* = 0.020) and aberrant cell frequency (*p* = 0.010) were independent predictors of maximal genitourinary toxicities in addition to V_100%_ and BED. As the GI toxicity frequency was low, we omitted any analysis of these toxicities in order not to overinterpret these results (5 Grade ≥ 2 toxicities).

## 4. Discussion

To our knowledge, there is no comprehensive analysis of T-lymphocyte DNA damage after different radiotherapy modalities. We found that the T-lymphocyte damage did not clear from the blood over a period of nine months to five years (depending on the type of treatment). The frequency of damaged cells in the case of teletherapies exceeded 10% at the three-month mark and remained at approximately 5% after one to five years. The difference among radiotherapy techniques is interesting because we could measure the summarized effect of dose rate, overall treatment time, energy, fractionation, and other parameters, which differ between the techniques and are difficult to calculate.

The chromosome aberration method is performed on lymphocytes, which are induced to divide by phytohemagglutinin M (PHA). According to the literature, in peripheral blood, only T-lymphocytes are sensitive to PHA [22]. One implication of our study is the possible impact of radiotherapy on the efficacy of immunotherapy; however, it was not addressed directly in our study. However, it was shown by others that T-cell DNA damage have the ability to cause senescence [4], from which this immune surveillance may be impaired [6], an immunosuppressive phenotype may develop [5], and defects in immunotherapy efficiency might follow [7,8,9]. Larger prospective studies with comprehensive clinical follow-up are required to establish whether the less observed aberrations in case of brachytherapies translate into meaningful clinical benefit in case of subsequent immunotherapy.

Studying lymphopenia was not one of our goals, but we examined the available blood test data and found only 19 lymphocyte counts under 1000/µL in 690 measurements (2.7%). Others have also described rare decreases in patients without pelvic irradiation [33,34]. One can speculate that immunological effects of radiotherapies, which do not affect bone marrow or pelvic lymph nodes, might not be due to a decrease in lymphocyte count. Our work suggests that the use of brachytherapy may hinder immunotherapy less than teletherapies. However, further studies are needed to investigate which immune cell subpopulations are affected.

The lifetime of lymphocytes remains a subject of debate in the literature. For instance, one study estimated the lifespan of lymphocytes to be 530 ± 64 days in a cohort of 25 women, whereas an upper limit of 29 months has also been proposed [35]. In another study, chromosome aberrations—which are typically lost during cell division—were still detectable in CD45RA+ T cells ten years after radiotherapy [36]. Furthermore, studies in mice and humans have demonstrated that naïve T cells can exhibit the longest lifespans [37,38]. Memory T cells were also shown to have a long-lived dividing population that maintains immunological memory, but individual memory T cells are not especially long-lived [38,39]. In the present study, the inducing agent phytohemagglutinin M selectively stimulates T-lymphocytes [21,22,23,24], and therefore, the measurements reflect this cell population. Moreover, T-lymphocytes can migrate into lymph nodes, where they may be retained for extended periods [40,41]. Subsequent re-entry of these cells into the peripheral blood might contribute to an increase in detectable chromosome aberrations at later follow-up time points.

Few publications have applied the chromosome aberration technique, and even fewer have applied long-term follow-up to radiotherapy patients because the method is highly labor-intensive. However, there are published studies with similar conclusions, mostly with shorter follow-up periods. Hille et al. reported significantly more chromosome aberrations one year after prostate LINAC radiotherapy than before treatment [14]. They also measured total aberration values after LINAC therapy, which were comparable to our results. Hartel et al. found that one year after radiotherapy for prostate cancer, not just translocations, which are considered long-persisting aberrations, but also non-transmissible aberrations detected by the FISH technique (dicentrics and non-transmissible exchanges) were more frequent than at baseline [42]. Previously, in highly exposed plutonium workers, complex chromosome aberrations persisted for decades [43]. Furthermore, non-cancer patients irradiated with previous radiotherapy techniques and analyzed via a highly similar chromosome aberration method presented higher aberration values approximately 20–40 years after irradiation than controls [44,45]. These studies included a greater number of patients (max. 143), but in these studies bone marrow could have been affected.

We analyzed the effects of baseline characteristics on chromosome aberrations (Supplementary Table S3) and identified time as the primary predictor, demonstrating a universal significant effect across all modalities. Following brachytherapy, T status and iPSA significantly influenced the frequency of aberrations (gamma models/intensity drivers), whereas neither variable significantly predicted the presence of aberrations (binomial model/gatekeeper).

The inclusion of treatment characteristics in the models eliminated the independent effects of Gleason score and T status. However, iPSA remained a significant predictor. In the cases of brachytherapies, ADT (androgen deprivation therapy) use emerged as a significant independent predictor of chromosome aberrations. Within the teletherapy groups, seminal vesicle dose acted as an independent predictor of aberration frequency. While the application of SIB technique significantly influenced the presence of aberrations after conventional LINAC therapy Supplementary Table S4).

We found that HDR therapy, which caused the fewest chromosome aberrations, also had fewer toxicities, but it also caused a slower decrease in PSA and shorter biochemical relapse-free survival (even if the patient number is insufficient for a firm conclusion). We suggest that the immune cell damage is associated with the total irradiated volume, and because of the technique of brachytherapy, it is smaller than in the case of teletherapy techniques. Supporting this, LDR brachytherapy also caused fewer chromosome aberrations than teletherapies, but is known to lead to similar or better biochemical progression-free and overall survival [46,47]. We previously analyzed isodose surface volumes of prostate LDR, HDR brachytherapy, and conventional LINAC therapy and found that isodose volumes of the same doses were the smallest in the case of HDR therapy but were comparable to those of LDR brachytherapy [48]. In this study, 19 or 21 Gy was given in one fraction as monotherapy, but since the start of our study, 2 × 13.5 Gy has been shown to be superior and to provide excellent tumor control [49]. This is clearly a limitation of our study. Accordingly, tumor control was insufficient in our HDR brachytherapy arm, because the tumor may not have been sufficiently irradiated. The cause may be that in the case of monotherapy, whole-gland coverage and the accurate dosage of microscopic extensions are more important. Furthermore, neither re-oxygenation nor cell-cycle redistribution can help in the tumor cell destruction [50].

Our analysis was also limited by the fact that we analyzed a limited number of conventional LINAC patients; however, patient preference shifted toward faster therapies. There was also an initial imbalance in IPSS values; the conventional LINAC and CyberKnife groups had a higher initial mean IPSS than the brachytherapy groups even after propensity score weighting.

An important limitation of this study is that there are different doses (e.g., 19–21 Gy in HDR group) and techniques (conventional or SIB plan in LINAC group) applied within the modality groups without sufficient numbers for subpopulation analyses. Therefore, the observed differences caused by radiation delivery technique cannot be separated from other effects which may independently influence clinical outcomes.

Lymphocytes were used as surrogate normal tissue markers and have been proposed as predictive radiosensitivity markers [11,12,13,51]. Therefore, we investigated the predictive value of chromosome aberrations for toxicities. T-lymphocyte chromosome aberrations do not cause toxicities but might serve as surrogate predictors. We corrected the analysis for the biologically effective dose (BED) and irradiated volume (V_100%_) and found that total aberration values and aberrant cell frequencies are independent predictors of maximal genitourinary toxicities. According to our previous publication, there is a direct correlation between in vivo aberrations measured after radiotherapy and aberrations in the in vitro-irradiated blood of these patients (taken before treatment) [52]. These results may suggest that the chromosome aberration assay performed on patient’s blood might be suitable for identifying radiosensitive patients. On the other hand, the method is not a high-throughput technique, and other predictive tests may be more likely to be introduced into clinical use [53]. It is important to note that radiological techniques have also been explored for monitoring treatment-related immune responses [54,55].

We analyzed nearly 192,000 cells individually under a microscope (192 patients, 10 samples each, at least 100 cells/sample). There are only a few chromosome aberration studies with long follow-up periods in the literature because the method is highly labor-intensive. We also described the effect of CyberKnife therapy, which has emerged as a new therapeutic modality, and there is a need to identify its niche in prostate treatment [56].

## 5. Conclusions

We found that compared with brachytherapy, teletherapy increased the frequency of T-lymphocyte chromosome aberrations by 1.6–3.6 fold after prostate radiotherapy. The proportion of affected T-lymphocytes reached approx. 10% in the first three months. In our study, radiation-induced chromosome aberrations in T-lymphocytes did not disappear (i.e., return to pre-treatment values) even five years after treatment. Further research into the subtypes of damaged T-lymphocytes is needed to assess their implications.

## Figures and Tables

**Table 1:** Baseline characteristics of the patients in our study.

	Conventional LINAC	Cyberknife	LDT BT	HDR BT	*p* Value
Patients	24	50	66	52	
Age (Mean ± SD)	68.2 ± 6.3	67.3 ± 5.9	67.0 ± 6.6	65.1 ± 5.4	0.070
iPSA (Mean ± SD)	10.9 ± 4.4	9.6 ± 3.6	9.6 ± 3.5	8.9 ± 3.0	0.600
T status (%)					
T1b	4.2	2.0	0.0	0.0	0.996
T1c	25.0	12.0	25.8	26.9	
T2a	29.2	42.0	33.3	30.8	
T2b	12.5	30.0	13.6	13.5	
T2c	29.2	14.0	27.3	28.8	
Gleason score (%)					
≤6	45.8	40.0	74.2	69.2	0.002
7	54.2	60.0	25.8	30.8	
Risk (%)					
low	16.7	16.0	22.7	42.3	0.009
interm.	83.3	84.0	77.3	57.7	
HT (%)					
HT0	12.5	74.0	31.8	50.0	<0.001
HT1	87.5	26.0	68.2	50.0	
conv./SIB	33.3/66.7	0/100	-	-	
19/21 Gy	-	-	-	36.5/63.5	

Patients characteristics are shown according to the different treatment groups. (iPSA = initial PSA, LDR BT = low dose rate brachytherapy, HDR BT = high dose rate brachytherapy, Cyber. = CyberKnife therapy, Conv. = conventional LINAC based teletherapy, Risk according to NCCN stratification, HT1 = ADT receiving, SIB = Simultaneous integrated boost.

**Table 2:** Baseline characteristics after propensity score weighting.

	Conventional LINAC	Cyberknife	LDT BT	HDR BT	*p* Value
Patients	23.2	44.4	64.5	50.4	
Age (Mean ± SD)	67.6 ± 5.8	66.8 ± 6.0	66.3 ± 7.1	66.1 ± 5.3	0.281
iPSA (Mean ± SD)	9.9 ± 3.2	10.1 ± 3.9	9.9 ± 4.0	9.6 ± 3.2	0.266
T status (%)					
T1b	0.7	0.7	0.0	0.0	0.939
T1c	20.2	24.7	22.3	18.4	
T2a	39.6	34.3	34.4	40.7	
T2b	11.4	19.8	17.2	17.6	
T2c	28.2	20.5	26.1	23.2	
Gleason score (%)					
≤6	70.6	58.1	60.5	59.3	0.449
7.00	29.4	41.9	39.5	40.7	
Risk (%)					
low	34.0	15.8	24.0	23.2	0.408
interm.	66.0	84.2	76.0	76.8	
HT (%)					
HT0	43.9	50.2	43.2	54.6	0.637
HT1	56.1	49.8	56.8	45.4	
conv./SIB	17.4/82.6	0/100	-	-	
19/21 Gy	-	-	-	23.1/76.9	

Patients characteristics are shown according to the different treatment groups. (iPSA = initial PSA, LDR BT = low dose rate brachytherapy, HDR BT = high dose rate brachytherapy, Cyber. = CyberKnife therapy, Conv. = conventional LINAC based teletherapy, Risk according to NCCN stratification, HT1 = ADT receiving, SIB = Simultaneous integrated boost.

**Table 3:** Models predicting cumulative late genitourinary toxicities.

	Chromosome Breaks			Total Aberrations			Aberrant Cells			Dicentrics + Rings		
	*p*	Beta	Beta SD	*p*	Beta	Beta SD	*p*	Beta	Beta SD	*p*	Beta	Beta SD
Model (*p*)	0.003			0.000			0.000			0.001		
Model (R2)	0.078			0.106			0.112			0.088		
Model (Cohan f2 effect size)	0.084			0.118			0.126			0.097		
BED (*p*)	0.003	−0.232	0.077	0.000	−0.291	0.079	0.000	−0.303	0.079	0.001	−0.260	0.078
V_100%_ (*p*)	0.040	0.156	0.075	0.634	0.042	0.089	0.897	0.012	0.093	0.535	0.062	0.100
aberration (*p*)	0.621	0.039	0.078	0.020	0.219	0.093	0.010	0.253	0.098	0.145	0.151	0.103

Multivariate linear regression models for cumulative late genitourinary toxicity are shown with the following variables: BED (biological effective dose) and V_100%_ (the volume irradiated with 100% of the prescribed dose) and chromosome aberrations directly after radiotherapy (chromosome breaks, total aberrations, aberrant cell frequency, dicentrics + rings frequency) as predictors. The *p*-value, the R2 (coefficient of determination) and the Cohen’s f2 effect size are given for the models, and *p*-values and beta coefficients (±SD) are shown for the individual variables.

## Data Availability

The datasets presented in this article are not readily available because the data are part of an ongoing study. Requests to access the datasets should be directed to kocsis.zsuzsa@oncol.hu.

## Supplementary Materials

The following supporting information can be downloaded at: https://www.mdpi.com/article/10.3390/cancers18162536/s1, Figure S1: PSA decrease (A) and biochemical relapse free survival (B) as a result of conventional LINAC (black) and CyberKnife (gray) therapy, LDR BT (burgundy) and HDR BT (red) (A). The significant differences from post-hoc tests compared with the PSA values after conv. LINAC therapy are indicated with asterisks * in the appropriate color. The error bars represent the SEM. Table S1. Dose volume constraints. Table S2: Post-hoc tests. Table S3: Effect of baseline characteristics on chromosome aberrations. Table S4: Effect of baseline and treatment characteristics on chromosome aberrations.
