# Supplementary material for: Radiotherapy Technique Determines the Magnitude and Persistence of T-Lymphocyte DNA Damage
Source: Cancers (Basel). 2026 Aug 7;18(16):2536. doi: 10.3390/cancers18162536 (PMC13511478; doi:10.3390/cancers18162536)
Supplement: Supplementary file 1 [file cancers-18-02536-s001.zip › Figure S1.pdf]

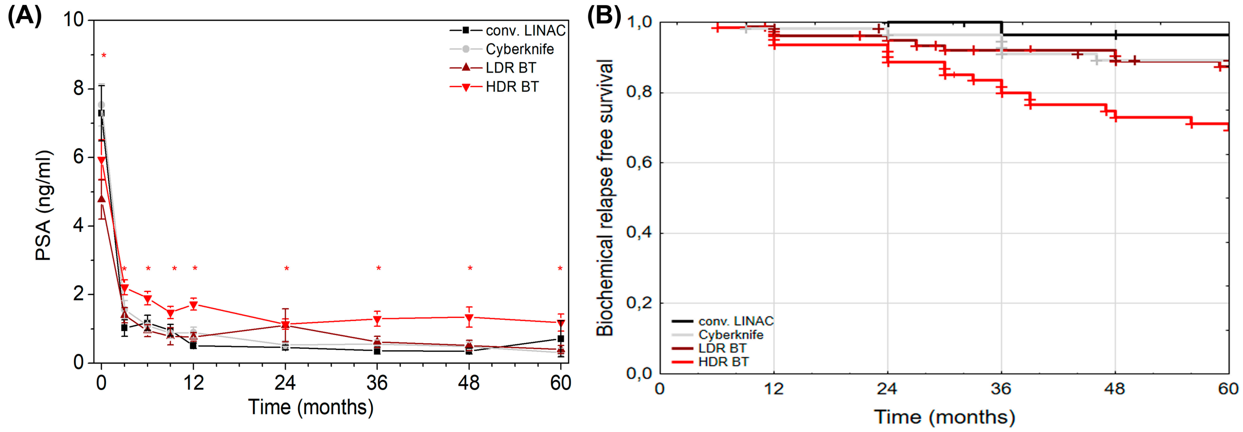

Figure S1. PSA decrease (A) and biochemical relapse free survival (B) as a result of conventional LINAC (black) and CyberKnife (gray) therapy, LDR BT (burgundy) and HDR BT (red) (A). The significant differences from post-hoc tests compared with the PSA values after conv. LINAC therapy are indicated with asterisks * in the appropriate color. The error bars represent the SEM.
